# Analysis of the current situation and factors influencing bullying in junior high schools in backward areas of Western, China & A case study of Qingyang City in Gasu

**DOI:** 10.1186/s12889-024-18775-5

**Published:** 2024-05-13

**Authors:** Hongjing Li, Chunyuan Liu, Xiping Shen, Yingdong Nan, Liya Feng

**Affiliations:** 1 Department of Basic Medical Sciences, College of Medicine, Longdong University, Qingyang, 745000 Gansu China; 2 Young Pioneers Brigade, Dongjie Primary School in Zhenyuan County, Qingyang, 745000 Gansu China; 3 Epidemic and Statistics Teaching and Research Office, School of Public Health, Lanzhou University, Lanzhou, 730000 China

**Keywords:** Junior school student, School bullying, Risk factors, Regression analysis

## Abstract

**Background:**

Qingyang is located in the northwest of China. By analyzing the current situation and risk factors of bullying in junior high schools in Qingyang City, and identify relevant data for formulating prevention and control measures of bullying in western backward areas.

**Methods:**

Qingyang City is divided into four regions based on economic level and population quality. One junior high school is randomly selected from each region, a total of 1200 students from 4 junior high schools of different levels in Qingyang City were randomly selected, and the “Questionnaire on Middle School Students’ School bullying” was administered between December 2021 and February 2022.

**Results:**

The reporting rate of bullying in junior high schools in Qingyang was 47.35%. The incidence of campus bullying among urban-rural integration junior high schools, senior students, and male students is higher than that of municipal -level junior high schools, junior students, and female students (*P*< 0.05). The results of binary logistic regression showed that the second grade of junior high school (OR = 1.39,95% CI: 1.022–1.894), poor student performance (OR = 1.744,95% CI: 1.09–2.743), external dissatisfaction (OR = 2.09,95% CI: 1.177–3.427), mother working in an enterprise (OR = 1.623,95% CI: 1.074–2.453), and urban-rural integration middle school (OR = 3.631,95% CI: 2.547–5.177) were factors affecting bullying in junior high school campus.

**Conclusion:**

The reporting rate of bullying in junior high schools in Qingyang City was relatively high, mostly occurring in places lacking supervision and after-school hours.

**Trial registration:**

Not applicable.

**Supplementary Information:**

The online version contains supplementary material available at10.1186/s12889-024-18775-5.

## Background

Bullying on school campuses has been a prominent issue of concern in China [1]. Since the issuance of the “Notice on Implementing the Annual Action for Preventing and Combating Bullying among Primary and Middle School Students” by the Office of the State Council’s Education Supervision Committee in 2018, China [2], various provinces and cities have also introduced governance plans and targeted measures [3]. As a result, school bullying has been curbed and transformed to some extent. In recent years, with the development of new media (i.e., products and services that provide information or entertainment), the exposure to school bullying incidents has gradually increased [4], drawing widespread attention from various sectors of society due to the diversity of bullying forms, the covert nature of behaviors, and the severe consequences. As is well known, ongoing peer victimization may lead to a variety of adverse psychosocial outcomes, such as anxiety, avoidance, depression, isolation, poor confidence, lack of control, impaired concentration, and poor academic achievement, all of which may have further negative implications and repercussions in the professional and social life of the targeted individuals [5]. Middle school students, being the primary demographic affected by school bullying [6], have been the focus of extensive research in this field within the country. Although the scope of research is broad, there has been relatively limited in-depth investigation in less developed northwestern regions [7]. Furthermore, most previous studies are theoretical studies, while there is an obvious lack of empirical analyses [8].

According to one nationwide study based in China, 13.61% were victims of bullying [7]. This number was substantially higher in Xi’an Province, where a staggering 38.70% of adolescents experienced bullying at school [9]. As this inconsistency in the prevalence of bullying may be due to differences in the population’s composition, it is of utmost importance to collect regional-specific epidemiological data to completely understand the regional differences in the prevalence of bullying.

Qingyang is a prefecture-level city in the far east of Gansu Province located in a channel on the middle stretches of the Yellow River on the Loess Plateau in the northwest region of China. This city lags in economic development and has lower education and cultural development levels than the more developed eastern cities. It is a residence to a substantial number of migrant workers and left-behind children. In this study, we assessed the occurrence and risk factors of bullying in junior high schools in Qingyang City and identified relevant data for formulating prevention and control measures of bullying in western backward areas. Public awareness of campus bullying incidents mainly comes from the internet, as there is scarce scholarly research on the causes, consequences, and prevention measures of bullying [10]. We conducted a comprehensive survey on the current status of school bullying and its risk factors, hoping to identify effective measures for preventing and addressing school bullying and to provide relevant references for optimizing ideological and political education for students in the new era and improving school management systems.

## Methods

### Study setting

**Fig. 1:**
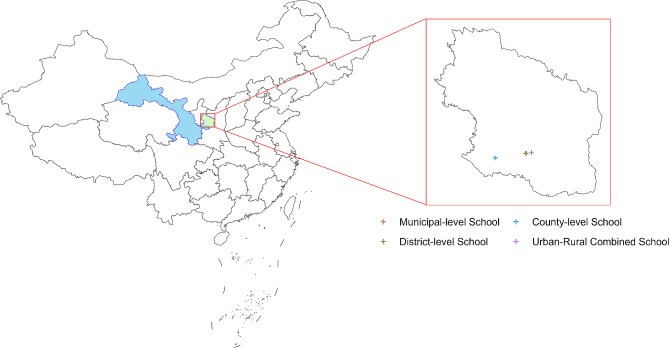
Map of study location. The blue color represents Gansu Province, and the green color represents Qingyang

Qingyang City encompasses seven county towns and one urban district, with junior high schools located in the primary urban areas of each county serving as the research subjects. As of the survey date, a total of 16 junior high schools were included. This study employed a phased sampling approach. In the first stage, based on geographical location and surrounding population, the research subjects were categorized into four levels (municipal -level, district-level, urban-rural fringe, and county-level junior high schools)**(**Fig.1**)**, comprising three municipal -level junior high schools, three district-level junior high schools, three urban-rural fringe junior high schools, and seven county-level junior high schools.Municipal-level schools are directly administered by the city’s education department and typically receive more investment and support in terms of faculty, educational facilities, and resources. District-level schools are under the direct jurisdiction of administrative education departments within their respective districts, with student populations distributed across various administrative regions. Resource allocation in these schools tends to emphasize local educational characteristics and positioning. Urban-rural combination schools are primarily located at the junction of urban and rural areas and often face challenges such as insufficient faculty, limited educational resources, and difficult teaching conditions. Left-behind children usually attend them. County-level schools are located in various counties and districts, with student populations usually coming from the county-level administrative area. While these schools may have relatively weaker subject offerings, teaching resources, and faculty strength, they prioritize establishing good social relationships and campus culture.

In the second stage, one school was randomly selected from each category of junior high schools. Using a proportional sampling method, 100 students from each grade were randomly selected, forming a sample. Such an approach ensured that the composition of students led by different grade-level teachers was the same, thus controlling for the influence of homeroom teachers on the survey results. Participants with significant mental illnesses requiring long-term treatment or those who have recently experienced physical or psychological trauma and did not consent to participate in this survey were excluded. The on-site questionnaire survey was conducted from December 2021 to April 2022.

### Questionnaire survey

For the purposes of the present study, we designed “Middle School Student School Bullying Survey Questionnaire” based on the relevant domestic research [5]. Initially, a preliminary survey was conducted with 50 seventh graders from a specific school using the draft questionnaire to understand their comprehension of instructions and items. Items that were difficult to understand or were ambiguous were modified or removed to create the final survey tool. The questionnaire covered four aspects: basic information, social support, bullying behaviors, and school measures, totaling 66 items (Supplementary material). The present study defined social support as the sum of prosocial behaviors from friends, family, teachers, and schools. Bullying was defined as a variety of situations where an individual was subjected to prolonged and repeated bullying or harassment by one or more individuals or was targeted as the victim of bullying. Campus bullying was defined as bullying perpetuated by students that typically occurred both inside and outside the school environment and included verbal bullying, physical bullying, relational bullying, and cyberbullying. Behaviors such as insults, ridicule, mockery, teasing, name-calling, and threats were defined as verbal bullying. Actions such as hitting, kicking, scratching, shoving, extortion, theft, and property damage were defined as physical bullying. Relational bullying typically encompassed aspects of verbal bullying, such as the spread of rumors affecting the victim being excluded or ostracized from a group. The deliberate hostile behavior toward peers using electronic media to harass, humiliate, or cause harm to others was defined as cyberbullying [11]. Social support, bullying behaviors, and school measures were evaluated on a Likert five-point scale, with higher scores indicating greater severity. Bullying behaviors encompassed verbal bullying, physical bullying, relational bullying, and cyberbullying, with a total of 12 questions. The scores ranged from 1 to 5 based on the frequency of bullying, with a total score range of 12 to 60. A score > 12 indicated that at least one type of school bullying had occurred, defining the respondent as a victim of school bullying. To ensure the reliability of the survey results, investigators were selected from grade-level class teachers with the support of the schools and informed consent from the participants. They received uniform training and were supervised by project researchers. An audit team conducted on-site reviews of the questionnaires, promptly addressing any issues.

The overall reliability of the questionnaire was 0.818, with Cronbach’s α coefficients for the social support, bullying behavior, and school measure dimensions of 0.819, 0.887, and 0.929, respectively, indicating good internal consistency.

The results of the Kaiser-Meyer-Olkin (KMO) test and Bartlett’s test of sphericity showed that KMO = 0.947 > 0.6, and the significance level of the sphericity test was*P*< 0.05. Therefore, the exploratory factor analysis was the suitable approach. The exploratory factor analysis divided the questionnaire items into three dimensions, with a cumulative variance contribution rate of 61.066%.

### Parameters definition

School Type: defined based on the school’s geographical location and its student body’s composition.

Academic performance: students ranked in the top 10% of the class were considered excellent, those ranked between 10% and 30% were considered good, those ranked between 30% and 70% were considered average, and those in the bottom 30% were considered poor.

Physical fitness: those with very good physical condition, rarely sick, actively participated in various sports activities and achieved good results were considered excellent; those with strong physiques, actively engaging in sports, rarely sick were considered good; those who were physically average for their age group, participated only in school-arranged physical exercises, and occasionally fell sick, which did not significantly affect their normal learning were considered average; those with weak physical condition, frequently sick, often on sick leave which affected their normal learning were considered poor.

Appearance satisfaction: was defined as subjective judgment, reflecting an individual’s confidence to a certain extent.

Single-parent status: was defined as living with only one parent due to divorce, death of one parent, separation, or other reasons.

Economic status: was categorized as follows: both parents having an income, monthly income exceeding 10,000 yuan, and no major illness sufferers in the family was considered as having relatively good economic status; both parents having an income, monthly income around 8,000 yuan, and no major illness sufferers in the family was considered as average economic status; one parent having income, monthly income below 5,000 yuan, and no major illness sufferers in the family was considered as barely sufficient for normal living; one parent having an income, monthly income below 5,000 yuan, and there are major illness sufferers in the family are considered economically challenging.

Understanding of bullying: regularly pays attention to relevant reports, lectures, videos, etc., on campus bullying, and having a certain understanding of how to avoid bullying were considered as a good understanding of bullying; curiously follows reports and videos on campus bullying, knows behaviors that constitute bullying but lacks awareness of how to prevent it were considered as having a general understanding of bullying; knows nothing about bullying was considered as having no understanding of bullying.

### Statistical methods

Following the EPV (events per variable) principle [12] and assuming a bullying occurrence rate of 40% [9], in this study, all independent variables were categorical variables, including dummy variables, totaling 36 independent variables included in the regression equation. When EPV = 10, the number of bullying incidents in the school was calculated as 36*10 = 360 cases, with a total sample size of 360 ÷ 40%=900 cases. Considering a 20% dropout rate in the sample, the minimum sample size to be included was calculated as 9001.2 = 1080 cases.

A double-entry database was established with EpiData 3.1 software (EpiData Association, Odense, Denmark) and IBM SPSS Statistics, Version 26.0 (IBM, Armonk, NY). Continuous data following normal distribution were expressed as mean ± standard deviation (𝑥̅ ± 𝑠), and multiple group comparisons were conducted using one-way analysis of variance (ANOVA). Categorical data were expressed as percentages (%), and intergroup comparisons were performed using the χ² test. Binary logistic stepwise regression analysis was conducted to identify the independent risk factors associated with bullying behavior in middle school students (Table S1). Multiple linear regression analyses were utilized to identify the association between social support and school bullying scores among middle school students. The entry and removal criteria were set at 0.05 and 0.10, respectively.

We hypothesized that social support would be associated with the bullying scores. R software(version 4.1.3) was used for the data analysis, the assessment of regression model was made by“performance” package. Our results indicated that verbal, physical, and emotional bullying and cyberbullying all passed the tests for influential points and VIF (Variance Inflation Factor) and generally passed the linearity test. However, they showed some limitations in the homoscedasticity of residuals and normality, possibly due to the presence of independent variables in the residuals that the model did not consider. Overall, these findings largely met the assumptions of multiple linear regression (Figures S1-S4).

## Results

A total of 1,200 subjects were included in the survey, with 1,134 valid questionnaires collected, resulting in an effective response rate of 94.50%. A total of 593 male students (52.29%) and 541 female students (47.71%) were assessed (see Table 1); 243 subjects were from municipal-level schools (21.43%), 313 from district-level schools (27.60%), 300 from urban-rural combined schools (26.46%), and 278 from county-level schools (24.51%); 365 students were from grade 7 (41.01%), 376 in grade 8 (28.74%), and 393 in grade 9 (30.25%). Other different demographic characteristics are shown in Table 1.

**Table 1:** Comparison of school bullying total scores among different demographic characteristics (𝑥̅ ± 𝑠, points)

Demographic Characteristic		Number	Bullying Total Score	t/F	*P*
Gender	Male	593	15.44 ± 0.26	5.657	0.018
	Female	541	14.63 ± 0.21		
Grade	Grade 7	465	14.59 ± 0.25	9.274	0.000
	Grade 8	326	14.56 ± 0.26		
	Grade 9	343	16.16 ± 0.37		
Academic Performance	Excellent	120	14.92 ± 0.58	2.575	0.053
	Good	405	14.58 ± 0.27		
	Average	491	15.22 ± 0.26		
	Poor	118	16.17 ± 0.51		
Physical Fitness	Very Good	154	14.95 ± 0.55	3.483	0.015
	Good	373	14.81 ± 0.29		
	Fair	548	15.00 ± 0.23		
	Poor	59	17.37 ± 0.78		
Self-Perceived Appearance Satisfaction	Very Satisfied	138	16.09 ± 0.71	5.806	0.001
	Satisfied	347	14.51 ± 0.27		
	Average	564	14.86 ± 0.22		
	Dissatisfied	85	16.92 ± 0.65		
Father’s Educational Background	College and Above	297	15.20 ± 0.40	2.896	0.034
	High School	366	14.38 ± 0.22		
	Middle School or Below	325	15.36 ± 0.30		
	Unknown	146	15.80 ± 0.57		
Father’s Occupation	Administrative/Corporate	217	14.93 ± 0.45	2.517	0.057
	Enterprise	283	15.51 ± 0.38		
	Service Industry	238	14.23 ± 0.28		
	Unemployed	396	15.30 ± 0.28		
Mother’s Educational Background	College and Above	219	15.13 ± 0.43	1.772	0.151
	High School	333	14.54 ± 0.29		
	Middle School or Below	425	15.16 ± 0.25		
	Unknown	157	15.77 ± 0.55		
Mother’s Occupation	Administrative/Corporate	163	14.56 ± 0.57	4.093	0.007
	Enterprise	232	15.38 ± 0.40		
	Service Industry	281	14.03 ± 0.22		
	Unemployed	458	15.35 ± 0.28		
Residence Status	Living with Both Parents	814	14.79 ± 0.19	2.333	0.072
	Living with Father	70	16.19 ± 0.72		
	Living with Mother	137	15.70 ± 0.54		
	Living with Other Relatives	113	15.52 ± 0.57		
Single-Parent Household	Yes	121	15.49 ± 0.55	0.762	0.383
	No	1013	15.01 ± 0.18		
Economic Status	Relatively Well-off	218	14.82 ± 0.38	0.928	0.427
	Surplus Income	355	14.89 ± 0.32		
	Sufficient for Basic Expenses	514	15.17 ± 0.25		
	Financial Difficulty	47	16.21 ± 0.74		
Boarding Status	Boarding	346	15.72 ± 0.30	3.410	0.033
	Non-Boarding	788	14.77 ± 0.21		
Awareness of School Bullying	Aware	710	15.16 ± 0.22	0.354	0.702
	Moderately Aware	380	14.86 ± 0.26		
	Not Aware	44	15.18 ± 1.13		
School Type	Municipal-level School	243	13.39 ± 0.22	24.947	0.000
	District-level School	313	17.11 ± 0.46		
	Urban-Rural Combined School	300	15.26 ± 0.25		
	County-level School	278	14.00 ± 0.27		

A double-entry database was established with EpiData 3.1 and IBM SPSS Statistics, Version 26.0

Continuous data following normal distribution were expressed as mean ± standard deviation (𝑥̅ ± 𝑠)

A*P*value < 0.05 indicates a statistical difference

### Comparison of School Bullying Total Scores among different Population groups

The total scores for school bullying behaviors demonstrated statistically significant differences among different populations, including gender, grade level, academic performance, physical fitness, self-perceived appearance satisfaction, father’s educational background, mother’s occupation, residence status (boarding status or residence status), and school type (*P*< 0.05, Table 1). Among these factors, male students, ninth-grade students, those with lower academic performance and physical fitness, those dissatisfied with their appearance, students with fathers of unknown educational background, students whose mothers worked in enterprises, boarding students, and students from district-level schools scored the highest. The total bullying score, to some extent, reflected the likelihood and severity of school bullying, suggesting that students with these characteristics were more likely to experience school bullying (Table 1).

### Distribution of school bullying incidence

The distribution of school bullying varied significantly among different schools and grade levels (*P*< 0.05). The results of the descriptive analysis indicated that urban-rural combined and district-level schools had a higher incidence of school bullying, i.e., 65.3% and 62.3%, respectively (Table 2). As grade levels increased, the incidence of school bullying followed, with the highest rate of 57.1% observed in Grade 9 (Table 2). There was no significant difference in school bullying incidence between genders (*P*> 0.05). However, the overall majority of middle school students have not experienced bullying, accounting for 52.64% of the population.

**Table 2:** Comparison of school bullying incidence by school, grade level, and gender (*n*, %)

Characteristics		Total Sample Size	Not Bullied	Bullied	χ^2^	*P*
School Type	Municipal-level School	243	167(68.7)	76(31.3)	103.119	< 0.001
	District-level School	313	118(37.7)	195(62.3)		
	Urban-Rural Combined School	300	104(34.7)	196(65.3)		
	County-level School	278	178(64.0)	100(36.0)		
Grade	Grade 7	365	199(54.5)	166(45.5)	10.813	0.001
	Grade 8	376	193(51.2)	183(48.8)		
	Grade 9	393	169(42.9)	224(57.1)		
Gender	Male	593	297(50.1)	296(49.9)	0.004	0.953
	Female	541	270(49.9)	241(50.1)		
Total		1134	597(52.64)	537(47.35)	-	-

A double-entry database was established with EpiData 3.1 and IBM SPSS Statistics, Version 26.0

Categorical data were expressed as percentages (%), and intergroup comparisons were performed using the χ² test

A*P*value < 0.05 indicates a statistical difference

The expression of bullying methods differed significantly among different schools, grade levels, and genders (*P*< 0.05). District-level schools and Grade 9 had higher scores for physical bullying, verbal bullying, relational bullying, and cyber bullying compared to other school types and grade levels (Table S2). Male students also more frequently experienced physical bullying and cyberbullying compared to female students, and these differences were statistically significant (*P*< 0.05) (Table S2).

### Multiple linear regression analysis of the impact of social support on school bullying behaviors

Multiple linear regression analyses were conducted using the scores for physical bullying, verbal bullying, relational bullying, and cyberbullying as dependent variables and scores for different types of support as independent variables (Table 3). The results revealed a negative correlation between social support and bullying behaviors. Specifically, family support and teacher support emerged as significant influencing factors for verbal bullying and physical bullying (all 0.05). For each additional point of family support, verbal bullying and physical bullying decreased on average by 0.087 and 0.049 points, respectively. For each additional point of teacher support, verbal bullying and physical bullying decreased on average by 0.141 and 0.109 points, respectively. Friend and teacher support significantly affected relational bullying (*P*< 0.05). For each additional point of friend and teacher support, relational bullying decreased on average by 0.062 and 0.089, respectively. Teacher support and school measures were identified as major influencing factors for cyberbullying (*P*< 0.05). For each additional point of teacher support and school measures, cyberbullying decreased on average by 0.091 and 0.042 points, respectively. The standardized regression coefficients indicated that teacher support had a greater impact on all four types of bullying behaviors. Based on these findings, it can be inferred that teachers, families, and friends have crucial roles in preventing and addressing school bullying in middle school settings (Table 4).

**Table 3:** Multiple linear regression analysis of factors influencing school bullying behaviors

Dependent Variable	Independent Variables	B	SE	Beta	t	Sig.	95%*CI*	
							Lower	Upper
Verbal Bullying	Constant	7.577	0.326		23.271	< 0.001	6.938	8.216
	Friend Support	-0.037	0.026	-0.051	-1.464	0.0143	-0.087	0.013
	Family Support	-0.087	0.025	-0.120	-3.448	0.001	-0.137	-0.038
	Teacher Support	-0.141	0.026	-0.184	-5.502	< 0.001	-0.191	-0.091
	School Measures	-0.011	0.006	-0.050	-1.718	0.086	-0.023	0.002
Physical Bullying	Constant	6.143	0.271		22.642	< 0.001	5.611	6.675
	Friend Support	-0.026	0.021	-0.042	-1.211	0.226	-0.067	0.016
	Family Support	-0.049	0.021	-0.081	-2.314	0.021	-0.090	-0.007
	Teacher Support	-0.109	0.021	-0.173	-5.112	< 0.001	-0.151	-0.067
	School Measures	-0.009	0.005	-0.055	-1.847	0.065	-0.020	0.001
Relational Bullying	Constant	5.848	0.285		20.509	< 0.001	5.289	6.408
	Friend Support	-0.062	0.022	-0.099	-2.791	0.005	-0.106	-0.019
	Family Support	-0.018	0.022	-0.029	− 0.810	0.418	-0.061	0.026
	Teacher Support	-0.089	0.022	-0.136	-3.981	< 0.001	-0.133	-0.045
	School Measures	-0.006	0.005	-0.031	-1.045	0.296	-0.016	0.005
Cyber bullying	Constant	5.306	0.244		21.703	< 0.001	4.826	5.786
	Friend Support	-0.036	0.019	-0.066	-1.861	0.063	-0.073	0.002
	Family Support	-0.007	0.019	-0.013	− 0.366	0.714	-0.044	0.030
	Teacher Support	-0.091	0.019	-0.161	-4.717	< 0.001	-0.128	-0.053
	School Measures	-0.042	0.018	-0.077	-2.358	0.019	-0.076	-0.007

A double-entry database was established with EpiData 3.1 and IBM SPSS Statistics, Version 26.0

Continuous data following normal distribution were expressed as mean ± standard deviation (𝑥̅ ± 𝑠), and comparison of influencing factors using linear regression

A*P*value < 0.05 indicates a statistical difference

**Table 4:** The location and time of bullying

	*N*(%)
Location	
Classroom	107 (21.0)
Corridor	79 (15.4)
Toilet	317 (61.9)
Playground	160 (31.4)
Campus corner	258 (50.5)
Around a school	253 (49.4)
Time	
During the class	34 (6.8)
During the break	243 (47.7)
After the class	338 (66.4)
Weekend or holidays	246 (48.3)

### Spatial distribution and coping mechanisms for school bullying

The locations where bullying victims experienced or witnessed school bullying were primarily concentrated in the restroom (accounting for 61.91%), school corners (accounting for 50.49%), and the vicinity of the school (accounting for 49.41%). In contrast, bullying in the school corridors was the lowest, at 15.43% (Table 4**)**. The temporal distribution of bullying incidents indicated that the majority of school bullying occurs after school hours (comprising 66.40% of cases), followed by during breaks between classes (accounting for 47.74%). Notably, 6.78% of school bullying incidents occured during classroom hours, which warrants attention.

After experiencing bullying, the majority of students choose to confide in their parents (comprising 61.12%) and teachers (accounting for 53.29%). A smaller percentage of students silently endured the situation (19.72%) or retaliated in response (16.25%). Among those who have experienced bullying, a significant proportion either report no psychological changes (26.81%) or experience feelings of inferiority (23.81%) (Table 5). Notably, 11.39% of students developed pessimistic and nihilistic emotions, and it is worth highlighting that 18.08% of students harbored resentment, which could represent a critical trigger for campus safety concerns.

**Table 5:** The solutions and psychological changes for bullying

		*N*(%)
Response	Bear	102 (19.7)
	Tell classmates	176 (34.0)
	Tell parents	316 (61.1)
	Tell teachers	275 (53.3)
	Call the police	139 (27.0)
	Counterattack	84 (16.3)
Psychological changes	Be self-abased	135 (23.8)
	Sad and world-weary	64 (11.4)
	Hate	102 (18.1)
	Worry and fear	84 (14.8)
	Remain unchanged	152 (26.8)

### Binary logistic stepwise regression analysis of risk factors for bullying behavior in middle school students

In this study, a binary logistic stepwise regression analysis was conducted to identify the independent risk factors associated with bullying behavior in middle school students (Table 6**)**. The occurrence of bullying behavior was used as the dependent variable, while sociodemographic characteristics that exhibited statistical significance in single-factor analysis were utilized as independent variables. The reference category was assigned to the lowest value for each independent variable. The results revealed that grade level, academic performance, self-perceived appearance satisfaction, mother’s occupation, and school type were the major risk factors contributing to the occurrence of bullying behavior among middle school students. The risk of experiencing bullying behavior in the second year of middle school (Grade 8) was 1.391 times higher than that in the first year (Grade 7) (*P*= 0.036). Students with good, average, and poor academic performance had respective risks of experiencing bullying behavior at 2.245, 2.108, and 1.744 times higher than those with excellent academic performance (*P*= 0.006, 0.002, 0.016). Individuals reporting moderate or low self-perceived appearance satisfaction had risks of experiencing bullying at 3.005, 2.103, and 2.009 times higher than those who were highly satisfied with their appearance (*P*= 0.001, 0.009, 0.011), respectively. The children of mothers employed in the corporate sector had a risk of experiencing bullying behavior 1.623 times higher than the children of mothers working in administrative positions (*P*= 0.022) (Table 6). Furthermore, the risk of bullying was 2.942 times higher in district-level combined urban-rural middle schools and 3.631 times higher in city-level middle schools compared to county-level middle schools (*P*= 0.000). These findings suggest that higher grade levels, lower academic performance, lower self-perceived appearance satisfaction, and attendance at district-level combined urban-rural middle schools are associated with an increased likelihood of experiencing bullying behavior. Conversely, students with mothers working in administrative positions are less likely to experience bullying.

**Table 6:** Binary logistic stepwise regression analysis of factors influencing bullying behavior in middle school students

Dependent Variable	B	SE	Waldχ^2^	df	Sig.	OR	95%CI	
							Lower	Upper
Grade			4.644	2	0.098			
Grade 8	0.330	0.157	4.405	1	0.036	1.391	1.022	1.894
Grade 9	0.108	0.172	0.392	1	0.531	1.113	0.795	1.559
Academic Performance			10.880	3	0.012			
Good	0.809	0.296	7.486	1	0.006	2.245	1.258	4.007
Average	0.746	0.238	9.856	1	0.002	2.108	1.323	3.357
Poor	0.556	0.231	5.795	1	0.016	1.744	1.109	2.743
Self-Perceived Appearance Satisfaction			11.955	3	0.008			
Satisfied	1.100	0.319	11.909	1	0.001	3.005	1.609	5.614
Average	0.743	0.283	6.903	1	0.009	2.103	1.208	3.662
Not Satisfied	0.697	0.273	6.546	1	0.011	2.009	1.177	3.427
Mother’s Occupation			9.059	3	0.029			
Enterprise	0.484	0.211	5.274	1	0.022	1.623	1.074	2.453
Service Industry	-0.039	0.184	0.044	1	0.834	0.962	0.671	1.380
Unemployed	0.172	0.170	1.023	1	0.312	1.187	0.851	1.657
School Type			85.070	3	0.000			
District-level School	1.079	0.171	39.838	1	0.000	2.942	2.104	4.112
Urban-Rural Combined School	1.290	0.181	50.781	1	0.000	3.631	2.547	5.177
County-level School	0.383	0.205	3.498	1	0.061	1.467	0.982	2.193
Constant Term	-0.952	0.361	6.938	1	0.008	0.386		

A double-entry database was established with EpiData 3.1 and IBM SPSS Statistics, Version 26.0

Categorical data were expressed as percentages (%), and comparison of influencing factors using logistic regression

A*P*value < 0.05 indicates a statistical difference

## Discussion

In this study, we assessed the occurrence and risk factors of bullying in junior high schools in Qingyang City and identified relevant data for formulating prevention and control measures of bullying in western backward areas. A total of 1200 students from 4 junior high schools of different levels in Qingyang City were randomly assessed using a questionnaire [5]. The reported prevalence of school bullying was 47.35%, which is lower than the findings from PISA 2015 but higher than that reported by Wang et al. [13] and Liu et al. [14] in Dalian, Shandong, China. Yet, our data are similar to reports by Shen et al. [15], who assessed rural areas of southern Henan.

Adolescent bullying may take many forms, such as verbal, relational, social or physical [16]. Verbal bullying (e.g., teasing in a hurtful way) and physical bullying (e.g., kicking, hitting, pushing, etc.) are usually considered to be direct forms. Relational bullying refers to indirect bullying, such as spreading rumors and social exclusion. Cyberbullying is the use of technology to harass, threaten, embarrass, or target another person. In terms of the manifestations of school bullying, in this study, the frequency of occurrence, from highest to lowest, was verbal bullying (40.7%), relational bullying (28.7%), physical bullying (28.4%), and cyberbullying (17.2%). These results are generally consistent with the findings reported by Ru et al. [17] in Jiangxi Province; however, the prevalence of various forms of bullying was much higher than in the study conducted by Yang et al. [18] in a certain region of central China. This indicates that, relative to eastern urban areas, the prevalence of school bullying in Qingyang City is closer to that of northern rural areas. Given that the research area is located in the northwest of the country, it is possible that the prevalence of school bullying in this region is influenced by parenting styles and factors such as economic conditions and educational attitudes, which have already been recognized as influential factors [19]. In economically disadvantaged areas, parents often have lower levels of education, and they tend to focus solely on their children’s academic achievements while neglecting their psychological well-being [20]. They may not know how to properly guide their children through sensitive psychological phases. Children who do not feel safe and secure within their families may be more inclined to seek warmth and care from their peers, making them more susceptible to joining groups involved in school bullying [21]. The higher prevalence of school bullying in urban-rural combined and district-level middle schools compared to city-level middle schools in this survey supports this perspective. Regarding the forms of school bullying, verbal bullying, relational bullying, and physical bullying remained prevalent. However, the relatively higher prevalence of cyberbullying compared to other cities suggests that students in less developed areas may be more influenced by harmful online information.

Differing from many domestic studies, the prevalence of school bullying did not show a significant difference between male and female students in this survey (*P*< 0.05), which may be related to the sample selection process and could also indicate that the dominant role of females in school bullying is gradually emerging. Interestingly, several school bullying cases reported in the surveyed area on the internet revealed that both bullies and victims were females, which is a noteworthy observation [22, 23]. However, in this study, male students scored significantly higher in terms of physical bullying and cyberbullying compared to female students (*P*> 0.05), which could be associated with the nature of male students, characterized by a higher level of physical activity, curiosity, and a preference for the virtual world, as has been confirmed by several previous studies [24]. Both the prevalence of school bullying and different bullying types increased from Grade 7 to Grade 9. This phenomenon can be largely attributed to the current educational philosophy in China.

The prevailing cultural emphasis in schools, as well as among parents and society, is placed on academic achievement as the primary indicator of a student’s worth [25]. Consequently, striving for academic success has become the mainstream culture within school environments. In such a climate, as students progress in grades and face increasingly challenging curricula, some students who struggle with their studies, achieve lower grades, or exhibit more introverted personalities may find it challenging to establish a sense of belonging and achievement within the mainstream school culture. They may be drawn to subcultures within the school that revolve around violence, bullying, or other deviant behaviors. Some scholars refer to this phenomenon as the influence of a school subculture [26]. In this study, regression analysis on the impact of social support on school bullying behavior revealed that family support has a significant role in verbal and physical bullying. Friend support was a independent influencing factor in relational bullying and cyberbullying. School measures were the independent influencing factor in cyberbullying, and teacher support had an impact on various forms of bullying. This highlights the need for relevant authorities to recognize the vital role of teachers in preventing and intervening in school bullying and to fully leverage teachers’ agency to effectively curb the occurrence of school bullying.

After controlling for the influences of gender, physical fitness, father’s education level, and residential status, the main risk factors for school bullying among middle school students were grade level, academic performance, self-perceived appearance satisfaction, mother’s occupation, and school type. Specifically, Grade 8 students (second-year middle school), those with poor academic performance, low self-perceived appearance satisfaction, students attending sub-city-level middle schools, and students whose mothers worked in the corporate sector constituted high-risk groups for school bullying. The reasons for this may be related to the critical importance of Grade 8, as it is a pivotal year for improving academic performance, especially for some struggling students. Failing to achieve satisfactory grades this year may result in an unfavorable outcome in the high school entrance examination (zhongkao). The expectations of their families and personal concerns about their future can impose significant psychological stress. If students lack self-confidence, they may seek validation through participation in school bullying, which is one of the reasons why some victims eventually become bullies [27]. Our results also suggested that good educational resources, student quality, and the mother’s occupational background positively impacted keeping students away from school bullying. Children whose mothers work in administrative departments are less likely to experience school bullying compared to those in the corporate sector. This may be because families in administrative departments often possess a certain social status and stable financial resources, prioritize family education and the transmission of values, and set stricter standards for child education and behavior, thereby reducing the likelihood of being bullied. Additionally, such families pay more attention to their children’s academic pursuits and well-being, ensuring that children are more likely to receive support and assistance from their family when facing challenges, which lessens the sense of isolation during times of bullying [28].

In this survey, the primary locations for school bullying were areas with limited supervision, such as restrooms, school corners, and the vicinity of the school. Bullying incidents were mainly reported during the time after school, which is consistent with the results of many previous studies [15, 17]. Encouragingly, most students who experienced bullying chose to confide in their parents and teachers, while a minority silently endured the situation or engaged in retaliation. The investigation into the psychological changes experienced by those who have been bullied reveals that a significant proportion either report no psychological changes or feel a sense of inferiority. This suggests that some students may adopt an indifferent attitude toward school bullying. Research indicates that considering bullying behavior as normal is a risk factor for perpetrating harm to others [29]. Therefore, it is recommended that parents and schools pay close attention to the psychological changes in children who do not exhibit emotional fluctuations after experiencing bullying and provide proper guidance.

The present study has certain limitations: (1) Lack of Unified Measurement Standards: the survey questionnaire designed for this study lacks unified measurement standards for reference. Developing a standardized assessment system specifically for school bullying is an urgent issue that needs to be addressed in the future. (2) Sample Selection: the selection of survey participants was based solely on school levels, without considering the actual educational quality and student quality, which may not be directly related to the school’s level. This factor contributes to the inconsistency of some survey results with most domestic reports. (3) Cross-Sectional Nature: this study is a cross-sectional survey, which means it cannot reveal the underlying causes of school bullying. (4) Results derived from different questionnaires cannot be directly compared. (5) Finally, we failed to offer more detailed information about bullying behaviors, such as the exact timeframe related to bullying behavior. (6) In this study, binary logistic stepwise regression analysis was conducted to identify the independent risk factors associated with bullying behavior in middle school students. Yet, stepwise regression analysis alone is not fully appropriate for causal inference. Future large-sample, multi-center prospective studies are warranted as they could enable a more rigorous analysis of the issue.

## Conclusion

To sum up, the reported rates of school bullying in Qingyang City were higher than those in the developed eastern cities and were similar to those in the western rural areas. Verbal bullying and physical bullying continued to be the main forms of local school bullying, while the incidence of cyberbullying was higher than that of other areas in China, and the incidence of school bullying seemed to be gradually rising with the increase in grades. Grade, achievement, appearance satisfaction, father’s occupation, and school type were the main factors affecting school bullying.

While verifying the important role of school, family, and society in school bullying in middle schools, this survey reflects the new trend of school bullying in the information age to a certain extent and has a positive role in enriching research data and conclusions on school bullying in backward areas in western China. Our findings can provide a theoretical basis for seeking a feasible policy of education and correction between “protection” and “punishment” of minors in the face of bullying.

### Electronic supplementary material

Below is the link to the electronic supplementary material.

Supplementary Material 1

Supplementary Material 2

Supplementary Material 3

Supplementary Material 4

Supplementary Material 5

Supplementary Material 6

## Data Availability

All data generated or analysed during this study are included in this published article.

## Abbreviations

ANOVA: Analysis of variance
PISA: Programme for International Student Assessment”
OECD: Organization for Economic Co-operation and Development
