# Supplementary material for: Analysis of the current situation and factors influencing bullying in junior high schools in backward areas of Western, China & A case study of Qingyang City in Gasu
Source: BMC Public Health. 2024 May 13;24:1295. doi: 10.1186/s12889-024-18775-5 (PMC11089733; doi:10.1186/s12889-024-18775-5)
Supplement: Supplementary file 1 — Supplementary Material 1 [file 12889_2024_18775_MOESM1_ESM.docx]

Survey Questionnaire on Bullying Behaviors Among Middle School Students

**Dear Student, **

**Greetings! In order to gain a deeper understanding of bullying behaviors among middle school students on campus, we have organized this survey. You have been selected to participate in our survey, and the results will be solely used for later data compilation and analysis. The situations listed in this questionnaire are those you may encounter during your time at school or while traveling to and from school. Please read them carefully and mark the appropriate box with a check mark (√). Your answers are neither right nor wrong, but they are highly important. We assure you that any answers you provide will be kept strictly confidential and will not be disclosed to teachers or fellow students. Please feel free to respond truthfully.**

**Note: Unless otherwise indicated, all questions below are multiple-choice questions, meaning only one option can be selected for each question.** 

**1、Basic Information (Please tick or fill in the answers you consider to be true)**

1.gender：

Ａ.maleＢ.female 

2.grade：

Ａ.grade7Ｂ.grade8Ｃ.grade9Ｄ.grade10Ｅ.grade11Ｆ.grade12

3. Academic Performance：

Ａ.excellentＢ.goodＣ.averageＤ.poor

4. Physical Fitness：

A. excellent B. above average C. average D. below average E. very poor

5. Satisfaction with Appearance

A. very satisfied B. satisfied C. neutral D. dissatisfied E. very dissatisfied

6. Father's Educational Background？

A. Postgraduate or above B. Bachelor's Degree C. College Diploma D. High School E. Junior High School or below F. Unclear

7. Father's Occupation？

A. Government or Public Sector B. State-owned Enterprise or Large Private Enterprise C. Service Industry D. Self-employed E. Farmer F. Unemployed

8. Mother's Educational Background？

A. Postgraduate or above B. Bachelor's Degree C. College Diploma D. High School Diploma E. Junior High School or below F. Unclear

9. Mother's Occupation？

A. Government or Public Sector B. State-owned Enterprise or Large Private Enterprise C. Service Industry D. Self-employed E. Farmer F. Unemployed

10. Living Arrangements：

A. With Both Parents B. With Father C. With Mother D. With Grandparents E. With Other Relatives 

11. Single-Parent Family：

Ａ.yesＢ.no

12. Family Economic Status：

A. Very Good B. Surplus C. Able to Meet Basic Expenses D. Difficulty Meeting Basic Expenses

13. Boarding Situation：

Ａ.boardingＢ.non-boarding

14. Awareness of Campus Bullying？

A. aware B. somewhat aware C. completely unaware

**2、Social Support**

15. During School：

（1）My friends can genuinely help me.

□1 always □2 often □3 sometimes □4 occasionally □5 never 

（2）When I encounter difficulties, my friends care about and understand me.

□1 always □2 often □3 sometimes □4 occasionally □5 never 

（3）When I am happy or sad, I am willing to share with my friends.

□1 always □2 often □3 sometimes □4 occasionally □5 never 

（4）My family willingly assists me in making various decisions.

□1 always □2 often □3 sometimes □4 occasionally □5 never 

（5）My family can provide tangible and concrete help. 

□1 always □2 often □3 sometimes □4 occasionally □5 never 

（6）When I encounter difficulties, I discuss with my parents. 

□1 always □2 often □3 sometimes □4 occasionally □5 never 

（7）In school, my teachers care about and encourage me. 

□1 always □2 often □3 sometimes □4 occasionally □5 never 

（8）When I or classmates encounter difficulties, teachers try their best to help.

□1 always □2 often □3 sometimes □4 occasionally □5 never 

（9）When there are conflicts among classmates, teachers handle them promptly and fairly.

□1 always □2 often □3 sometimes □4 occasionally □5 never 

（10）When I see violent scenes in movies, games, etc., I unconsciously want to imitate.

□1 always □2 often □3 sometimes □4 occasionally □5 never 

16. If you are bullied outside, the approach your parents tell you to take is：

Ａ. Fight back immediately, never suffer losses. Ｂ. Temporarily give in, seek revenge later.

Ｃ. Retreat, then tell the teacher, parents, or police. Ｄ. Have not communicated with parents.

17. Do you think harassment, insults, defamation, or public disclosure of personal information on the Internet will be legally accountable？

Ａ. yes Ｂ. no Ｃ. Uncertain 

If you think no, the reason is：

Ａ. Group mentality, difficult to hold individual responsibility.Ｂ. Internet behavior is not regulated by law.

Ｄ. Freedom of speech. Ｃ. Anonymity makes it difficult to trace.Ｅ. Joking, not to be taken seriously.

Ｆ. Other (please specify reasons)

**3、Bullying Behaviors**

18. During school, have you seen classmates: (Multiple choice) 

ＡA. Being laughed at or teased by others

B. Being threatened or intimidated with violence

C. Personal belongings deliberately damaged by other classmates

D. Robbed or extorted of belongings by others

E. Being excluded or isolated by other classmates

F. Personal privacy spread on the internet by others

G. Being verbally or physically sexually harassed by others

H. Have not seen classmates being bullied 

19. When you see someone being bullied at school or outside, what do you think？

A. This is wrong and should be stopped

B. They are just joking around, no malicious intent

C. The unfortunate person must have done something to deserve it

D. That's their business, not mine

E. Have not seen classmates being bullied 

20. During school, have you ever experienced：

（1）Classmates threatening me to buy things, do things, or do homework for them.。

□0 times □1-2 times □3-5 times □6-10 times □ more than10 times 

（2）Personal belongings hidden or maliciously damaged by classmates.

□0 times □1-2 times □3-5 times □6-10 times □ more than10 times 

（3）Classmates forcing me to give money or provide items.

□0 times □1-2 times □3-5 times □6-10 times □ more than10 times 

（4）Being physically assaulted by classmates with brooms, sticks, knives, etc.

□0 times □1-2 times □3-5 times □6-10 times □ more than10 times 

（5）Being assaulted by multiple classmates.

□0 times □1-2 times □3-5 times □6-10 times □ more than10 times 

（6）Being threatened or assaulted by outsiders due to disputes with classmates. 

□0 times □1-2 times □3-5 times □6-10 times □ more than10 times 

（7）Being given derogatory nicknames or verbally insulted by classmates (e.g., "crazy").

□0 times □1-2 times □3-5 times □6-10 times □ more than10 times 

（8）Being threatened or intimidated with malicious language by classmates (e.g., warned "be careful after school," "asking for a beating").

□0 times □1-2 times □3-5 times □6-10 times □ more than10 times 

（9）Classmates intentionally provoking me with pushing or jostling.

□0 times □1-2 times □3-5 times □6-10 times □ more than10 times 

（10）Being maliciously ostracized or excluded by classmates. 

□0 times □1-2 times □3-5 times □6-10 times □ more than10 times 

（11）My relationship with classmates being maliciously undermined.

□0 times □1-2 times □3-5 times □6-10 times □ more than10 times 

（12）Classmates spreading privacy or rumors about me on the internet.

□0 times □1-2 times □3-5 times □6-10 times □ more than10 times 

（13）Classmates making sexual harassment such as telling dirty jokes, sexual invitations, etc.

□0 times □1-2 times □3-5 times □6-10 times □ more than10 times 

（14）Being secretly watched (peeped) by classmates. 

□0 times □1-2 times □3-5 times □6-10 times □ more than10 times 

（15）Being touched inappropriately on sensitive areas by classmates.

□0 times □1-2 times □3-5 times □6-10 times □ more than10 times 

（16）Receiving vulgar language, pornographic images, videos, etc., sent by classmates through social media.

□0 times □1-2 times □3-5 times □6-10 times □ more than10 times 

**Note: The options listed in question 20 represent specific manifestations of bullying. For students who selected "0 times" for all options from (1) to (16), please proceed to question 28 and beyond. For others, continue answering the following questions.**

21. After being bullied, what changes did you experience psychologically？

A. Feeling inferior B. Feeling sad and pessimistic C. Developing hatred D. Feeling worried and scared E. No change 

22. How might you react after experiencing bullying at school? (Multiple choice)

A. Endure silently B. Tell classmates C. Tell parents D. Tell teachers E. Report to the police F. Retaliate

23. Where did you experience/observe bullying at school? (Multiple choice)

A. Classroom B. Corridor C. Restroom D. Playground E. Schoolyard corner F. School surroundings

24. When did you experience/observe bullying at school? (Multiple choice)

A. During class B. During breaks C. After school D. Weekends or holidays

25. When you were bullied, how did your classmates react：

A. Tell school security or teachers B. Watch and gossip C. Step forward to stop it D. Join in bullying E. Pretend not to see 

26. What actions did teachers take after knowing you were being bullied? 

A. Severely criticize the bullies B. Make the bullies write self-reflections C. Make the bullies apologize to you D. Ignore such incidents E. Teachers did not know 

27. How did your parents react after knowing you were being bullied：

A. Take you to school to find teachers B. Let you solve it yourself C. Seek explanation from the other party's parents D. Transfer you to another school E. Parents did not know

28. In your opinion, what type of students are most likely to be bullies at school：

A. Classmates B. Older students C. Younger students D. Strangers

29. In your impression, what characteristics do students who bully others typically have? (Multiple choice)

A. Physically strong B. Aggressive temper C. Good family background D. Good academic performance E. Poor academic performance F. Good interpersonal relationships G. Poor interpersonal relationships H. Other (please specify)

30. In your impression, what characteristics do students who are bullied typically have? (Multiple choice)

A. Physically weak or flawed B. Unattractive appearance or body shape C. Good academic performance D. Poor academic performance E. Good family background F. Poor family background G. Poor interpersonal relationships H. Weak personality

31. If you see a classmate being bullied at school, what actions might you take? (Multiple choice)

A. Step forward to stop it B. Tell a teacher C. Report to the police D. Watch and gossip E. Leave the scene

**4、Evaluation of School Measures**

32. Please evaluate the following aspects of your school

（1）Provision of psychological counseling courses. 

□ none □ 1 per semester □ 1-2 times per month □ 1 per week □ several times per week

（2）Provision of life education courses.

□ none □ 1 per semester □ 1-2 times per month □ 1 per week □ several times per week

（3）Provision of legal education courses.

□ none □ 1 per semester □ 1-2 times per month □ 1 per week □ several times per week

（4）Offering courses or lectures on self-protection. 

□ none □ 1 per semester □ 1-2 times per month □ 1 per week □ several times per week

（5）Organizing sessions to study school rules and regulations. 

□ none □ 1 per semester □ 1-2 times per month □ 1 per week □ several times per week

（6）Holding themed class meetings or activities on friendly student interactions. 

□ none □ 1 per semester □ 1-2 times per month □ 1 per week □ several times per week

（7）Holding themed class meetings or activities on proper internet usage and online safety. 

□ none □ 1 per semester □ 1-2 times per month □ 1 per week □ several times per week

（8）Emphasizing the prevention and control of campus bullying during meetings. 

□ none □ 1 per semester □ 1-2 times per month □ 1 per week □ several times per week

（9）Displaying special bulletin boards on preventing campus bullying. 

□ none □ 1 per semester □ 1-2 times per month □ 1 per week □ several times per week

（10）Conducting routine inspections to prevent campus bullying.

□ none □ 1 per semester □ 1-2 times per month □ 1 per week □ several times per week

**Thank you for your support and cooperation!** 
