# Supplementary material for: Analysis of the current situation and factors influencing bullying in junior high schools in backward areas of Western, China & A case study of Qingyang City in Gasu
Source: BMC Public Health. 2024 May 13;24:1295. doi: 10.1186/s12889-024-18775-5 (PMC11089733; doi:10.1186/s12889-024-18775-5)
Supplement: Supplementary file 2 — Supplementary Material 2 [file 12889_2024_18775_MOESM2_ESM.docx]

**Table S1**. Variable Assignment Table

| Factor | Variable Name | Assignment Description |
| --- | --- | --- |
| Gender | *X*_1_ | Male=1, Female=2 |
| Grade | *X*_2_ | Grade 7=1, Grade 8=2, Grade 9=3 |
| Academic Performance | *X*_3_ | Excellent=1, Good=2, Average=3, Poor=4 |
| Physical Fitness | *X*_4_ | Very Good=1, Good=2, Fair=3, Poor=4 |
| Self-Perceived Appearance Satisfaction | *X*_5_ | Very Satisfied=1, Satisfied=2, Average=3, Dissatisfied=4 |
| Father's Educational Background | *X*_6_ | Unknown=4, College or Higher=1, High School=2, Middle School or Lower=3 |
| Mother's Occupation | *X*_7_ | Administrative/Corporate=1, Enterprise=2, Service Industry=3, Unemployed=4 |
| Boarding Status | *X*_8_ | Boarding=1, Non-Boarding=2 |
| School Type | *X_9_* | Municipal-level School =1, District-Level School=2, Urban-Rural Combined School=3, County-Level School=4 |
| Bullying Occurrence | *Y* | No=0, Yes=1 |

**Table S2.** Comparison of School Bullying Method Scores by School Type, Grade Level, and Gender (𝑥̅ ± 𝑠, points)

| Characteristics | | Physical Bullying | Verbal Bullying | Relational Bullying | Cyber bullying |
| --- | --- | --- | --- | --- | --- |
| School Type | Municipal-level School | 3.30±0.09 | 3.60±0.09 | 3.30±0.06 | 3.19±0.07 |
|  | District-level School | 4.22±0.12 | 4.68±0.14 | 4.13±0.15 | 4.08±0.12 |
|  | Urban-Rural Combined School | 3.73±0.09 | 4.44±0.12 | 3.76±0.08 | 3.33±0.06 |
|  | County-level School | 3.48±0.08 | 3.81±0.11 | 3.44±0.07 | 3.27±0.06 |
| F | | 16.970 | 18.540 | 13.070 | 23.865 |
| P | | <0.001 | <0.001 | <0.001 | <0.001 |
| Grade | Grade 7 | 3.55±0.07 | 4.03±0.09 | 3.64±0.09 | 3.37±0.06 |
|  | Grade 8 | 3.62±0.09 | 4.07±0.11 | 3.58±0.08 | 3.29±0.07 |
|  | Grade 9 | 4.01±0.11 | 4.44±0.13 | 3.86±0.09 | 3.85±0.11 |
| F | | 8.386 | 4.392 | 2.487 | 14.887 |
| P | | <0.001 | 0.013 | 0.084 | <0.001 |
| Gender | Male | 3.86±0.07 | 4.28±0.09 | 3.69±0.08 | 3.61±0.07 |
|  | Female | 3.54±0.06 | 4.05±0.08 | 3.68±0.07 | 3.37±0.06 |
| t | | 10.522 | 3.614 | 0.031 | 7.309 |
| P | | 0.001 | 0.058 | 0.860 | 0.007 |

A double-entry database was established with EpiData 3.1 and IBM SPSS Statistics, Version 26.0

Categorical data were expressed as percentages (%), and intergroup comparisons were performed using the χ² test.

A P value < 0.05 indicates a statistical difference.
