# Supplementary figures and images for: Analysis of the current situation and factors influencing bullying in junior high schools in backward areas of Western, China & A case study of Qingyang City in Gasu
Source: BMC Public Health. 2024 May 13;24:1295. doi: 10.1186/s12889-024-18775-5 (PMC11089733; doi:10.1186/s12889-024-18775-5)

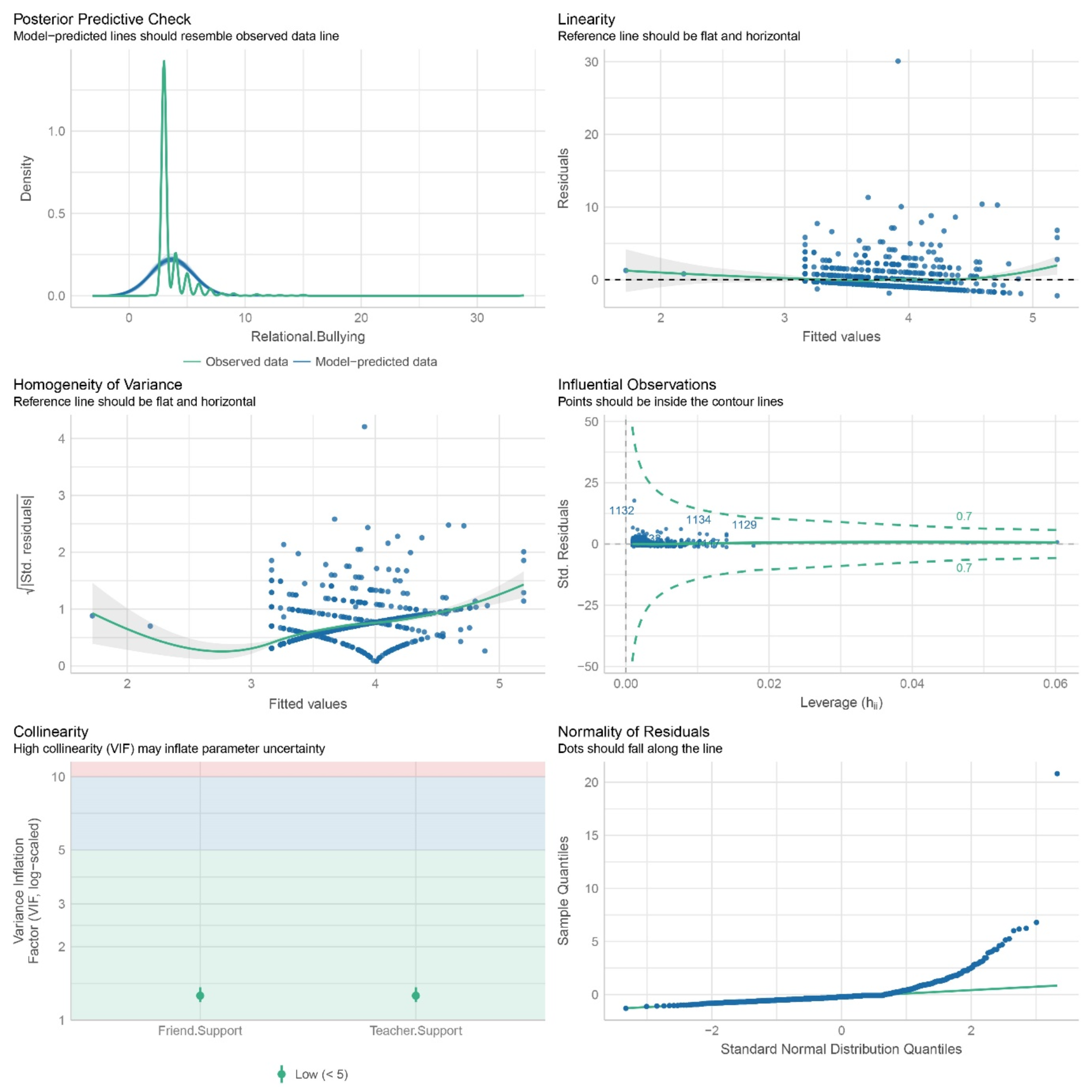

Supplement: Supplementary file 3 — Supplementary Material 3 [file 12889_2024_18775_MOESM3_ESM.tiff]

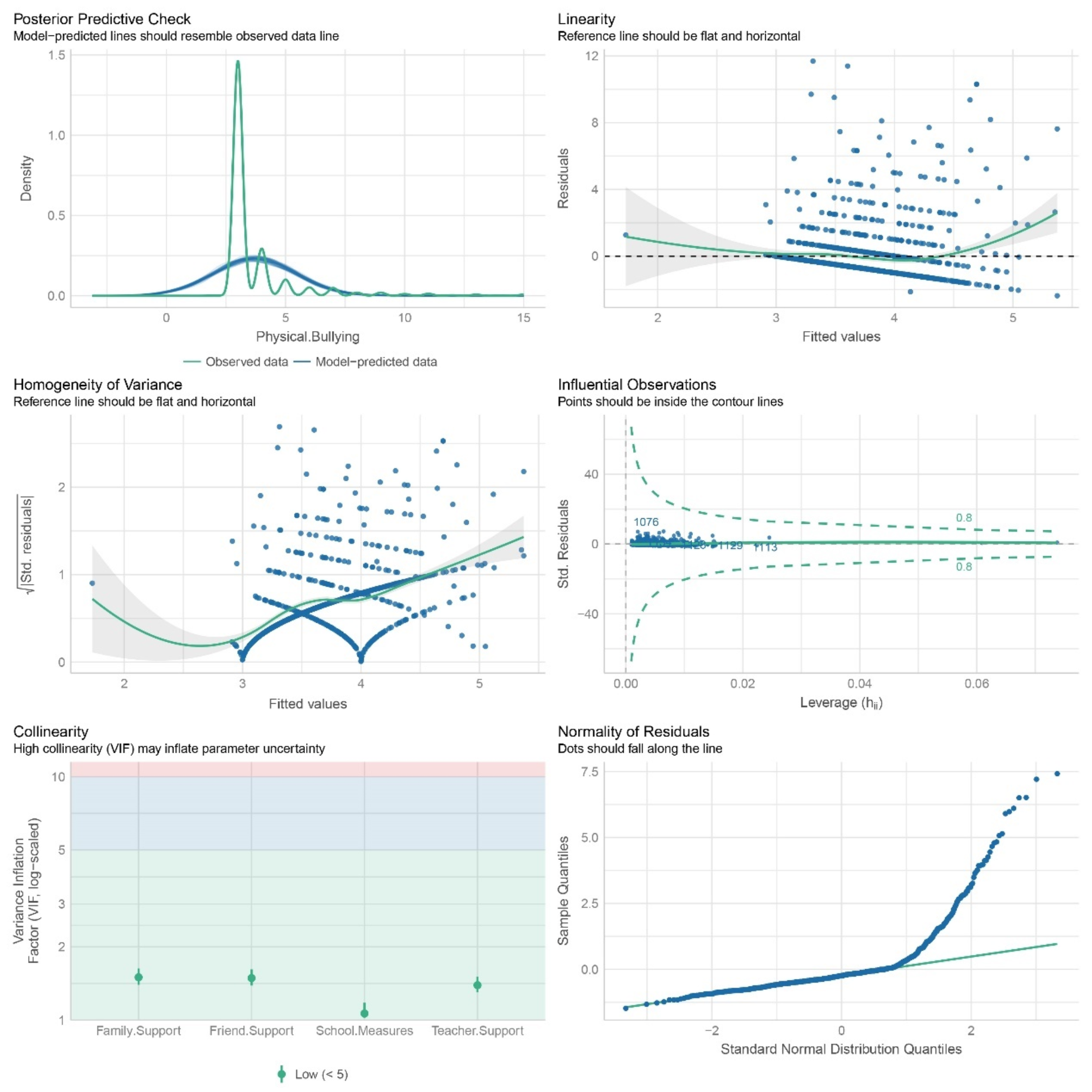

Supplement: Supplementary file 4 — Supplementary Material 4 [file 12889_2024_18775_MOESM4_ESM.tiff]

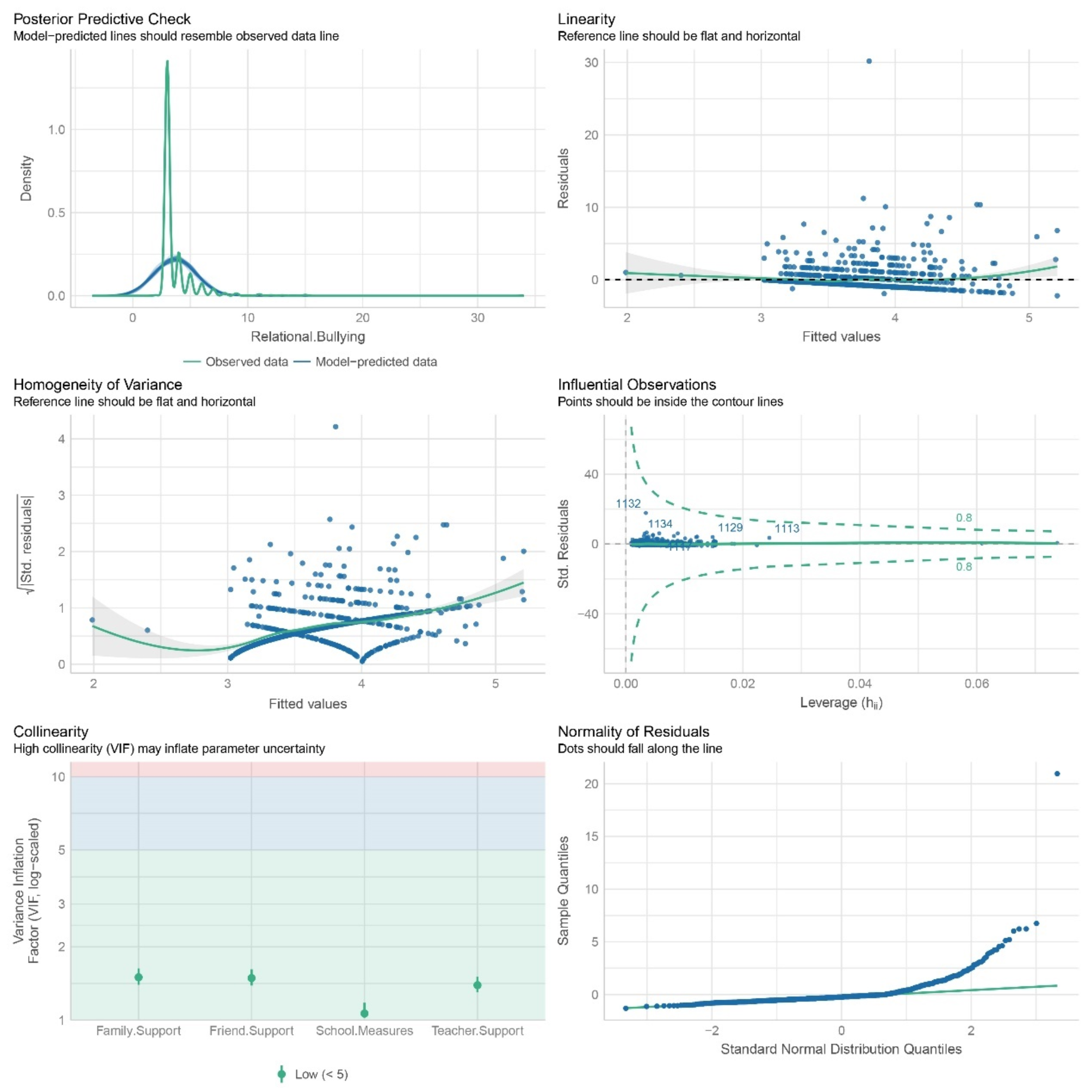

Supplement: Supplementary file 5 — Supplementary Material 5 [file 12889_2024_18775_MOESM5_ESM.tiff]

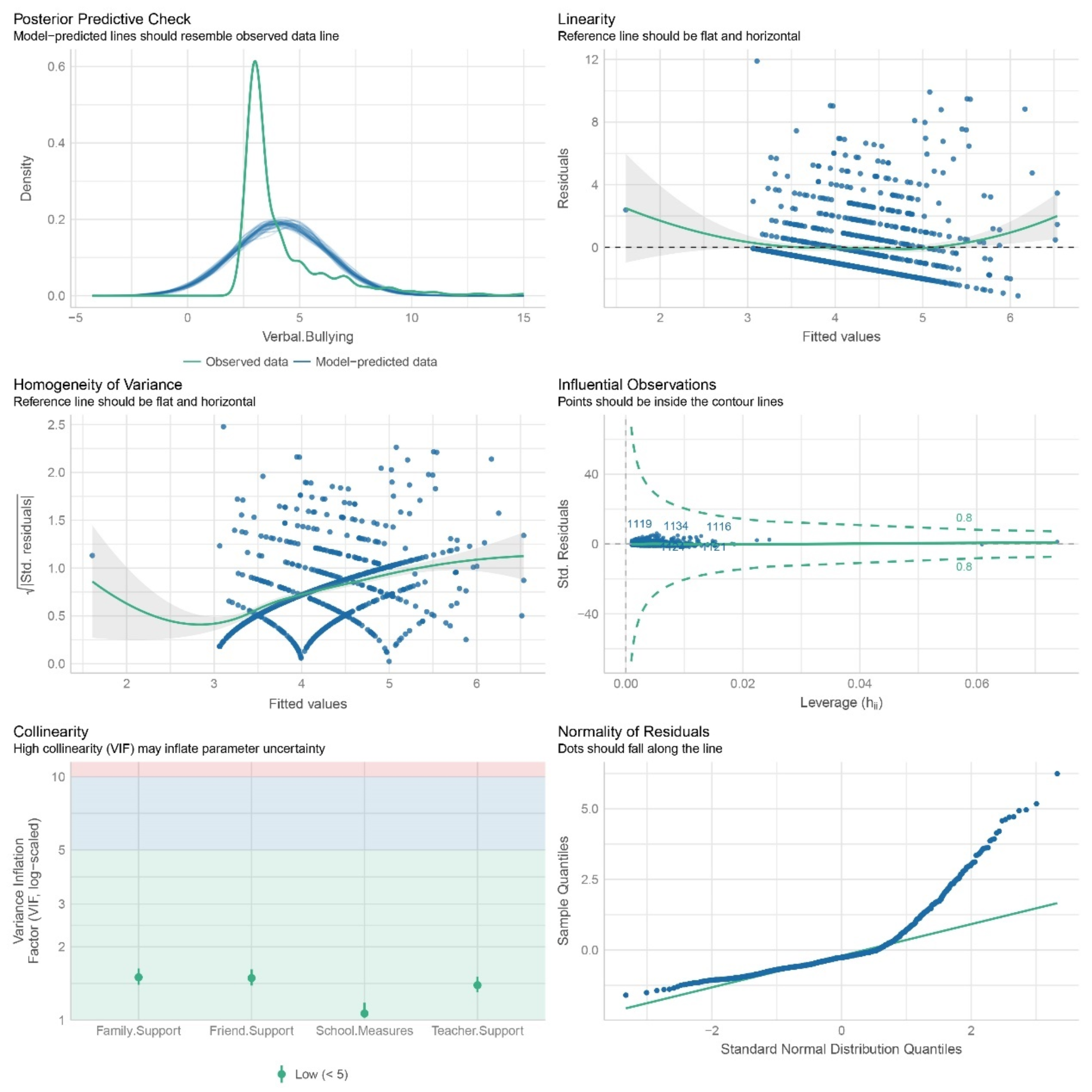

Supplement: Supplementary file 6 — Supplementary Material 6 [file 12889_2024_18775_MOESM6_ESM.tiff]
